# Improved Tolerance to Various Abiotic Stresses in Transgenic Sweet Potato (*Ipomoea batatas*) Expressing Spinach Betaine Aldehyde Dehydrogenase

**DOI:** 10.1371/journal.pone.0037344

**Published:** 2012-05-16

**Authors:** Weijuan Fan, Min Zhang, Hongxia Zhang, Peng Zhang

**Affiliations:** 1 National Key Laboratory of Plant Molecular Genetics, Institute of Plant Physiology and Ecology, Shanghai Institutes for Biological Sciences, Chinese Academy of Sciences, Shanghai, China; 2 Key Laboratory of Synthetic Biology, Institute of Plant Physiology and Ecology, Shanghai Institutes for Biological Sciences, Chinese Academy of Sciences, Shanghai, China; 3 Shanghai Chenshan Plant Science Research Center, Chinese Academy of Sciences, Shanghai Chenshan Botanical Garden, Shanghai, China; Kansas State University, United States of America

## Abstract

Abiotic stresses are critical delimiters for the increased productivity and cultivation expansion of sweet potato (*Ipomoea batatas*), a root crop with worldwide importance. The increased production of glycine betaine (GB) improves plant tolerance to various abiotic stresses without strong phenotypic changes, providing a feasible approach to improve stable yield production under unfavorable conditions. The gene encoding betaine aldehyde dehydrogenase (BADH) is involved in the biosynthesis of GB in plants, and the accumulation of GB by the heterologous overexpression of *BADH* improves abiotic stress tolerance in plants. This study is to improve sweet potato, a GB accumulator, resistant to multiple abiotic stresses by promoted GB biosynthesis. A chloroplastic BADH gene from *Spinacia oleracea* (*SoBADH*) was introduced into the sweet potato cultivar Sushu-2 via *Agrobacterium-*mediated transformation. The overexpression of *SoBADH* in the transgenic sweet potato improved tolerance to various abiotic stresses, including salt, oxidative stress, and low temperature. The increased BADH activity and GB accumulation in the transgenic plant lines under normal and multiple environmental stresses resulted in increased protection against cell damage through the maintenance of cell membrane integrity, stronger photosynthetic activity, reduced reactive oxygen species (ROS) production, and induction or activation of ROS scavenging by the increased activity of free radical-scavenging enzymes. The increased proline accumulation and systemic upregulation of many ROS-scavenging genes in stress-treated transgenic plants also indicated that GB accumulation might stimulate the ROS-scavenging system and proline biosynthesis via an integrative mechanism. This study demonstrates that the enhancement of GB biosynthesis in sweet potato is an effective and feasible approach to improve its tolerance to multiple abiotic stresses without causing phenotypic defects. This strategy for trait improvement in sweet potato not only stabilizes yield production in normal soils in unpredictable climates but also provides a novel germplasm for sweet potato production on marginal lands.

## Introduction

Sweet potato (*Ipomoea batatas*) is a major root crop that ranks seventh in annual production worldwide and is grown in more than 100 countries as a valuable source of food, animal feed, and industrial raw material [1], [2], [3]. Because of its tolerance to a wide range of agroecological conditions, high yield potential, ease of cultivation, effective vegetative propagation and high nutritive value, the sweet potato is suitable for growth on marginal lands [2]. Recently, a strategy for producing energy from non-grain biomass resources using marginal lands to minimize the potential impact of bioenergy production on food security was developed in China [4]. A recent survey revealed an area of 34 million hm^2^ of marginal land available for growing bioenergy crops in China and, potentially, 45 million metric tons of liquid biofuels may be produced if 60% of this area is utilized [5], [6]. As one of the most promising bioenergy crops, the sweet potato plays an important role in the development of first-generation biofuels in China [7]. However, pests, viral diseases and environmental stresses, e.g. low temperature and drought, frequently restrict the increased production of the sweet potato in many areas of the world [8]. New sweet potato varieties with enhanced tolerance to multiple abotic stresses are desirable.

Although traditional breeding has significantly contributed to trait improvement in sweet potato in the last several decades, producing a novel germplasm with desirable traits remains difficult due to the high levels of male sterility, self and interspecific incompatibility, and hexaploid nature of the sweet potato, which results in the strong segregation of hybrid progenies and the loss of many valuable traits [9], [10]. As an alternative, genetic engineering offers great potential for improving the sweet potato or generating useful breeding materials with improved traits, such as increased starch content and nutritional value, virus and nematode resistance and salt tolerance, by the expression of native or foreign genes [11], [12]. Recently, we established an efficient *Agrobacterium tumefaciens*-mediated transformation system for several farmer-preferred cultivars of sweet potato [13], which provided a routine tool for genetic improvement via transgenesis and mediated the examination of the function of exogenous and endogenous genes in sweet potato.

Improving yield production and stability under stressful conditions is necessary to fulfill the food demand of the ever-growing world population [14]. Besides the successful commercialization of transgenic crops with many desirable traits, e.g., resistance to insect pests, viral diseases or pesticides [15], an improved tolerance to adverse environmental conditions has also been achieved [16]–[18]. For improving abiotic stress tolerance in plants through genetic engineering, many studies have primarily focused on transcription factors for gene regulation [19], [20] and genes that encode ion transport proteins [21], compatible organic solutes [22], [23], antioxidants [24], [25], heat-shock [26], [27] and late embryogenesis abundant proteins [28], [29]. Although many genes associated with plant responses to abiotic stress have been identified and characterized in *Arabidopsis* and rice, their functions remain to be verified in other crops.

Different abiotic stress factors may induce osmotic and oxidative stress in plants, leading to similar cellular adaptive responses, such as the accumulation of compatible solutes and the acceleration of reactive oxygen species-scavenging systems [30]. Compatible solutes, including proline, trehalose, and GB, have been detected in a wide variety of organisms [23]. GB is a fully *N*-methyl-substituted derivative of glycine. This quaternary ammonium compound is present in bacteria, animals, and higher plants, and functions by stabilizing the quaternary structure of complex proteins, membrane systems, enzymes, and photosynthetic machinery, such as PSII complexes and RuBisCo [31]–[34]. The exogenous application of GB confers tolerance to various abiotic stresses in plants and increases their yield under salt conditions [34], [35]. In general, the GB biosynthetic pathway starts with choline and progresses through a two-step dehydrogenation of choline and oxygenation of betaine aldehyde catalyzed by CMO and BADH, respectively [33]. Plants overexpressing the genes responsible for GB synthesis, e.g., *BADH*, have an enhanced tolerance to a wide range of abiotic stresses, including salt, heat, drought, and chilling, by the increased accumulation of GB in plant cells [36]–[40].

In this report, we used *Agrobacterium* to transform embryogenic suspensions of the cultivar Sushu-2 and develop transgenic sweet potato plants expressing the *BADH* gene from *Spinacia oleracea*. The expression of the *SoBADH* gene increased BADH activity and GB synthesis in these transgenic sweet potato plants, which subsequently improved their tolerance to multiple abiotic stresses by induction or activation ROS scavenging and the accumulation of proline.

## Results

### Molecular, biochemical and phenotypic characterization of the So*BADH* transgenic sweet potato

**Figure 1:**
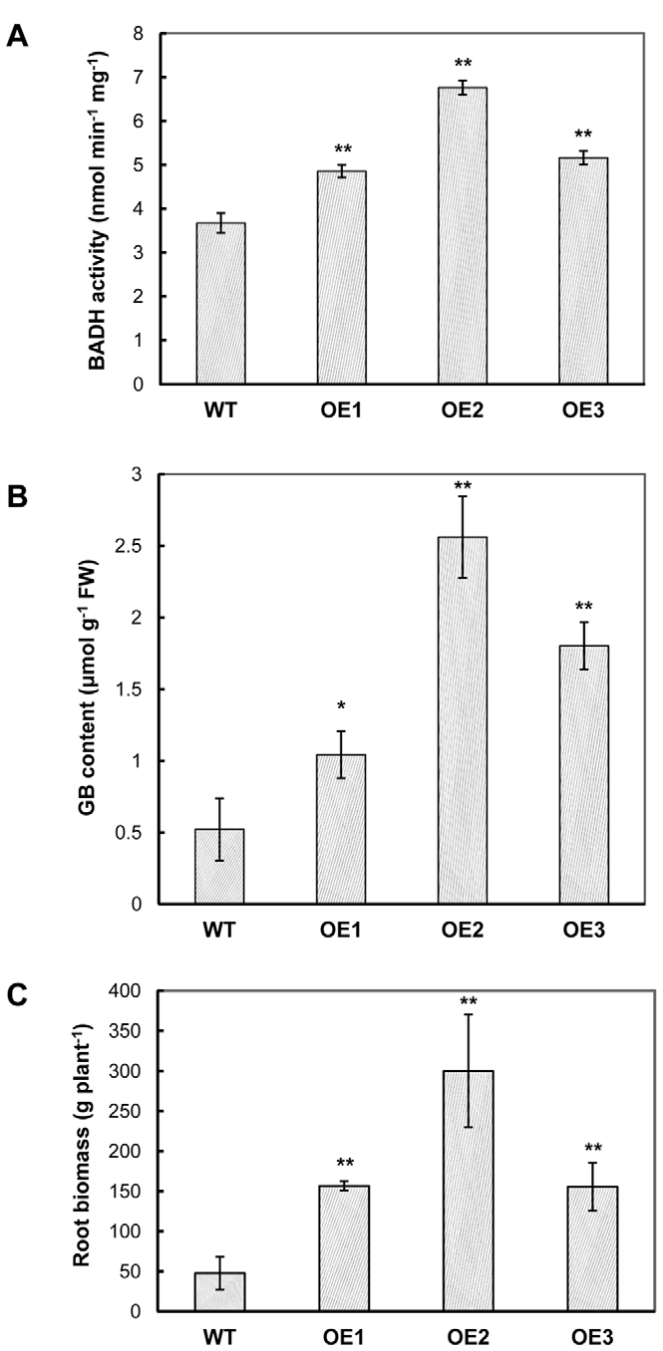
BADH accumulation and root biomass of wild-type and the *SoBADH* transgenic plants. A and B show the BADH activity and GB content in the leaves, respectively; C shows the root biomass. OE1–OE3, *SoBADH* overexpressing transgenic lines; WT, wild-type plant. Values represent the mean ± SD (n = 9). Asterisks indicate a significant difference from that of WT at * P<0.05 or ** P<0.01 by *t*-test.

Transgenic sweet potato plants overexpressing So*BADH* (termed OE) were successfully generated by the *Agrobacterium*-mediated transformation of the binary vector pCSoBADH (Figure S1) into embryogenic suspension cultures of the cultivar Sushu-2. More than 10 independent transgenic plant lines were established and propagated in the greenhouse. Most of these transgenic plant lines were phenotypically indistinguishable from the wild-type (WT) plants. After GUS staining, the transgenic OE plants displayed GUS activities in their leaves, in contrast with WT (Figure S2), confirming the stable integration and expression of the *uid*A gene. The integration of the pCSoBADH T-DNA into the genome of the transformed plants was determined by PCR analysis using *HPT*-specific primers (Table 1). Three single-integrated So*BADH* transgenic sweet potato lines OE1, OE2 and OE3 were verified in a Southern blot analysis by hybridizing *Eco*RI-digested genomic DNA samples with a So*BADH*-specific probe, and no integration was detected in the WT plants (Figure S3A). The RT-PCR analysis detected the expression of mRNA transcripts in all three OE lines but not in the WT line (Figure S3B), showing the expression of the So*BADH* gene in those transgenic plants. To further ascertain whether the So*BADH* overexpression is correlated with increased BADH enzyme activity, we measured the BADH activity and GB content in soluble extracts from the leaves of sweet potato plants (Figure 1A, B). The BADH activity and a GB content of 0.52 µmol g^−1^ were detected in the WT leaves, indicating that sweet potato is a natural accumulator of GB. Importantly, the BADH activity in the OE lines was elevated, and the OE2 plants exhibited a 1.84-fold increase in activity as compared with that of the WT plants. As a result, under the normal growth conditions, the GB content in the leaves of the OE lines was significantly higher than that of WT (Figure 1B). Plant line OE2 demonstrated the highest GB content at concentration of 2.56 µmol g^−1^, which is about a five-fold increase over that of WT.

**Table 1:** Phenotypic and primary molecular screening of the transgenic sweet potato lines from transformed cultivar Sushu-2.

Line	Rooting test*	Shoot phenotype	PCR	GUS staining	Insertion number
OE1	Rooted	normal	positive	stained	1
OE2	Rooted	normal	positive	stained	1
OE3	Rooted	normal	positive	stained	1
OE4	Rooted	normal	positive	stained	2
OE5	Rooted	normal	positive	stained	1
OE6	Rooted	normal	positive	stained	1
OE7	Rooted	normal	positive	stained	2
OE8	Rooted	normal	positive	stained	3
OE9	Rooted	normal	positive	stained	2
OE10	Rooted	dwarf	NT**	NT	NT
Sushu-2	-	normal	-	Non-stained	-

Notes:

* A rooting test was performed by culturing shoots on basic medium supplemented with 10 mg l^−1^ hygromycin and recorded after two weeks;

** Not tested.

When OE transgenic and WT plants were grown in the greenhouse under normal conditions, no visible phenotypic difference of their foliages was detected during the plant growth (Data not shown). After 5 months, the weights of storage roots from OE plant lines showed significant increase compared to those of WT plants. The yields of tuberous roots in the three OE plant lines were 156.67, 300 and 155.56 g plant^−1^, respectively, which is significantly heavier than that of the WT line (47.78 g plant^−1^) (Figure 1C).

### Enhanced tolerance of OE transgenic plants to high salinity stress

**Figure 2:**
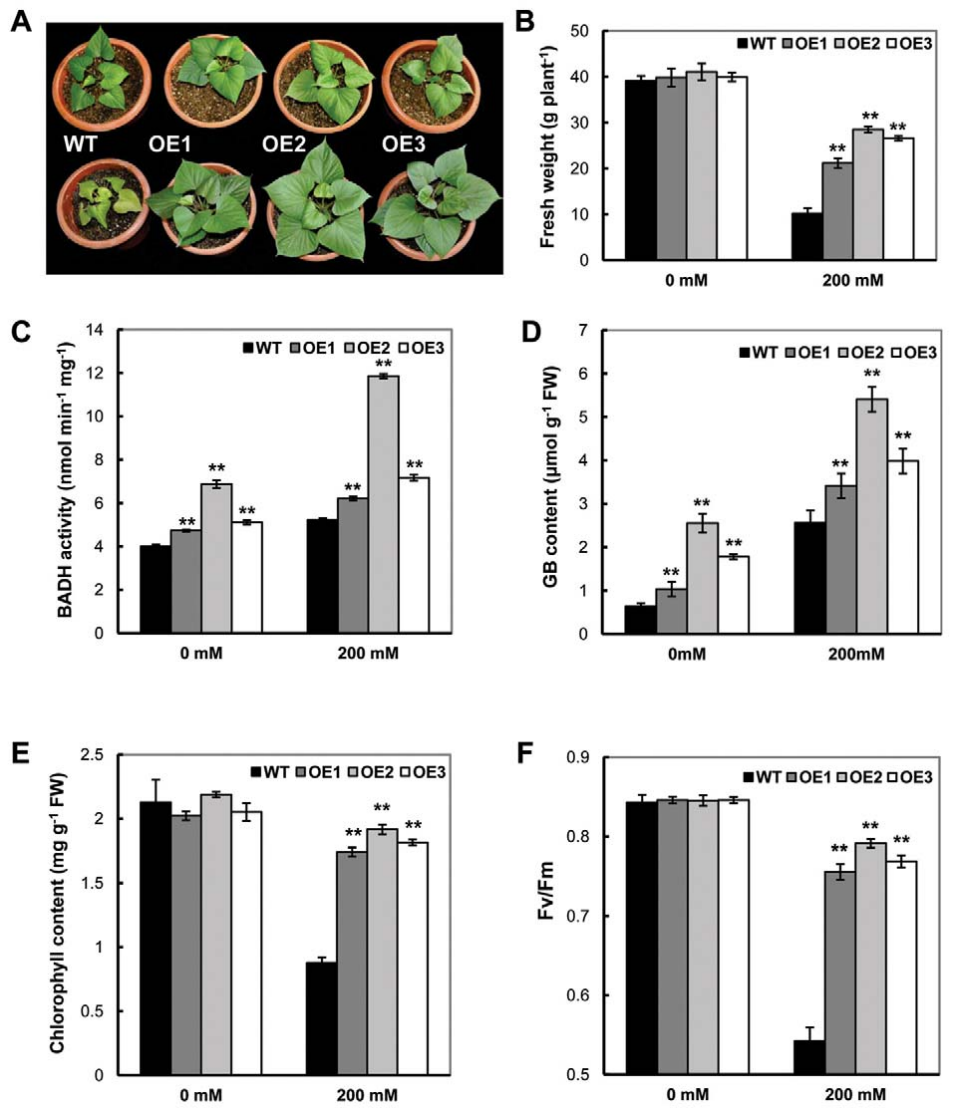
Phenotypic and physiological analyses of the *SoBADH* transgenic plants under 200 mM NaCl stress for 16 days. A, the phenotypic changes before (upper panel) and after NaCl treatment (lower panel); B, fresh weight of transgenic and WT plants after NaCl treatment; C, BADH activities in transgenic and WT plants before and after NaCl treatment; D, contents of GB in the leaves of transgenic and WT plants after NaCl treatment. E, chlorophyll content in the leaves of transgenic and WT plants after NaCl treatment; F, changes of maximum fluorescence ratios (Fv/Fm) in transgenic and WT plants upon NaCl stress. OE1–OE3, independent, *SoBADH* overexpressing transgenic lines; WT, wild-type plant. Values represent the mean ± SD (n = 9). Asterisks indicate a significant difference from that of WT at * P<0.05 or ** P<0.01 by *t*-test.

GB is primarily found in plants growing in salt-affected areas, and previous transgenic studies have demonstrated enhanced tolerance to salt stress in GB overexpressing lines [41]–[42]. In our study, after exposure to 200 mM NaCl for 16 days using 2-month-old plants, a significant impact on plant growth was observed (Figure 2A). Before treatment, the transgenic plants showed phenotypes indistinguishable from the WT plants (Figure 2A, upper panel); however, after treatment, the WT plants showed severely inhibited growth with yellowish leaf coloration (Figure 2A lower panel) and dramatically reduced fresh weight in contrast to the vigorous growth of OE plant lines (Figure 2B). We detected a reduction in the fresh weight of the transgenic plant lines from 40 g/plant to 25–30 g/plant (25%–37.5% reduction) as compared with 40 g/plant to 10 g/plant of reduction (74%) in the WT. The fresh weights of trangenic plants were a minimum of 2 times greater than WT plants under the stress treatment. The BADH activity and GB concentration in the leaves of the three OE transgenic plant lines were significantly higher than those of WT (*P*<0.05, Figure 2C, D). For example, in the WT, the GB content was increased five-fold from 0.63 µmol g^−1^ FW to 2.56 µmol g^−1^ FW after treatment. Upon treatment of the transgenic line OE2, the GB content was increased to 5.41 µmol g^−1^ FW in comparison with the 2.5 µmol g^−1^ FW of the untreated samples. Overall, the transgenic plant lines showed a 33.2% to 111.3% increase in GB content as compared with the WT after salt treatment. These results indicate that the enhanced tolerance to salt stress in the transgenic sweet potato is due to the increased accumulation of GB in plants.

The growth retardation observed in many plants under salt stress is often associated with a decrease in their photosynthetic capacity [43]. Under salt stress, the OE lines maintained a high level of chlorophyll content (11.6% to 13.9% reduction), while a dramatic decrease of 58.8% was detected in the WT (Figure 2E). To further evaluate the effects of stress-induced damage to photosystem II (PSII), the maximal efficiency of PSII photochemistry (Fv/Fm) was studied. Without salinity stress, there was little difference in the Fv/Fm ratio among these lines. After stress treatment, the transgenic OE lines maintained an 86–90% Fv/Fm ratio as compared with the 76% ratio maintained in WT (Figure 2F). These results indicate that the photosystem of OE transgenic plants is less affected than that of the non-transgenic WT plants during stress.

**Figure 3:**
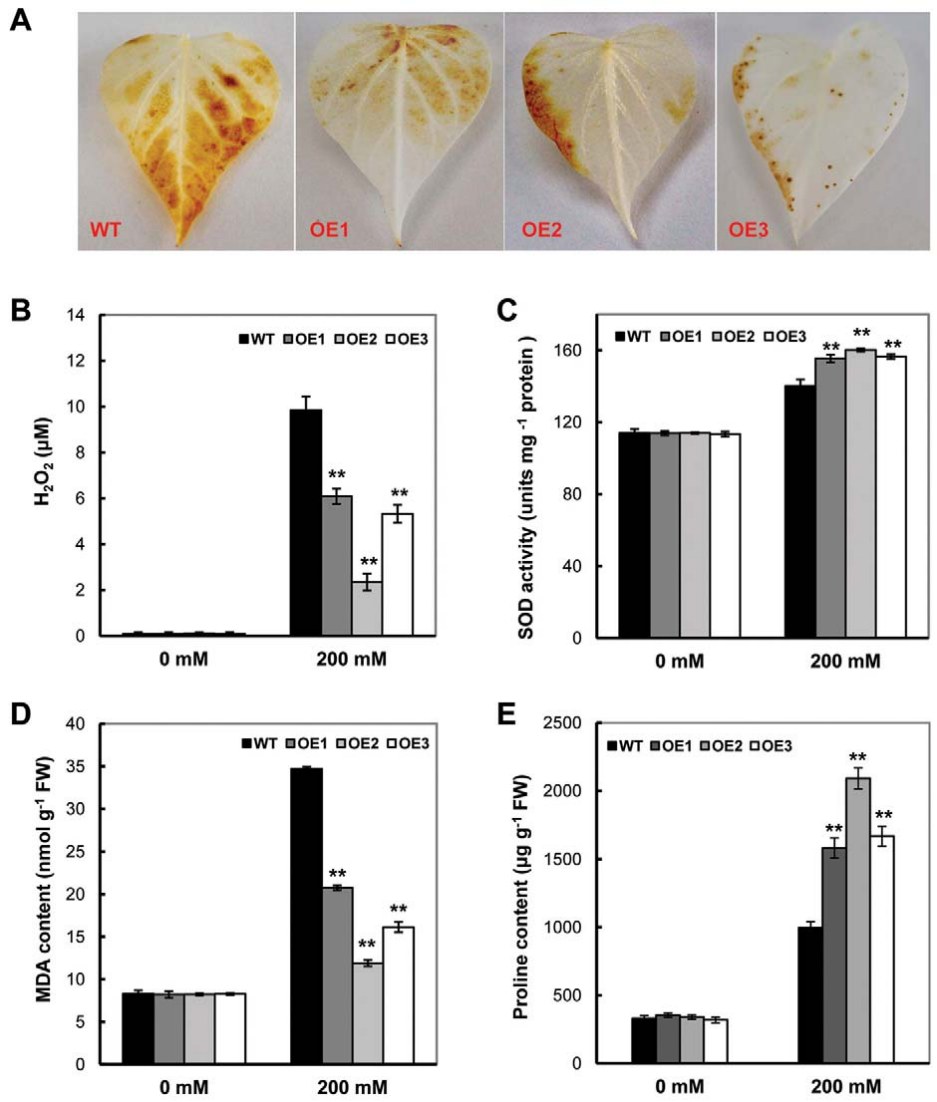
The *SoBADH* transgenic plants showed improved ROS scavenging and proline contents under 200 mM salt stress. A, detection of salt stress-induced H_2_O_2_ production by DAB staining in the leaves of the transgenic and WT plants; B–C, H_2_O_2_ and MDA contents in the leaves of the transgenic and WT plants; D, changes of SOD activity in transgenic and WT plants after NaCl treatment. E, proline contents in the leaves of the transgenic and WT plants. OE1–OE3, *SoBADH* overexpressing transgenic lines; WT, wild-type plant. Values represent the mean ± SD (n = 9). Asterisks indicate a significant difference from that of WT at * P<0.05 or ** P<0.01 by *t*-test.

Recent studies have demonstrated that elevated ROS inhibits the repair of photoinhibited PSII *in vivo* [43]. Therefore, we postulated that the accelerated repair of salt-enhanced photoinhibition induced by the accumulation of GB *in vivo* may be associated with the elevated ROS scavenging in transgenic plants. The salt stress stimulated an evident increase of H_2_O_2_ production in the leaves of the four sweet potato lines (Figure 3A, B). However, a significant decrease in H_2_O_2_ production, with 61.8%, 23.9% and 54.1% of the levels in WT, was observed in the transgenic lines OE1, OE2 and OE3, respectively (*P*<0.05) (Figure 3B). The SOD activity, an important indicator for ROS scavenging, in these plant lines was significantly increased after NaCl treatment, and the OE lines showed much higher activity than WT, with a 22.9% increase in WT and a 36.5% to 40.5% increase in the OE lines (Figure 3C). This difference indicated that the expression of the So*BADH* gene might protect the synthesis and/or activation of this enzyme, which results in less accumulation of ROS and cell damage in the transgenic plants. Similar to H_2_O_2_, NaCl treatment substantially increased the levels of malonaldehyde (MDA) in both the OE and WT lines (Figure 3D), but much less MDA accumulation was detected in the OE plants (Figure 3D), indicating decrease in lipid peroxidation under salt stress in the transgenic lines.

Under normal conditions, there was no significant difference in the proline content in the leaves of the transgenic OE lines and WT (Figure 3E). After 16 days of NaCl treatment, the proline content in the WT plants increased from 330 µg g^−1^ FW to 990 µg g^−1^ FW, which is a 3-fold difference. In the OE lines, however, the fold changes of proline content in the leaves was 4.46 to 6.14, which is much higher than that of the WT. For example, the proline contents of OE2 leaves were increased from 340 µg g^−1^ FW before the treatment to 2091 µg g^−1^ FW after the treatment (Figure 3E).

**Figure 4:**
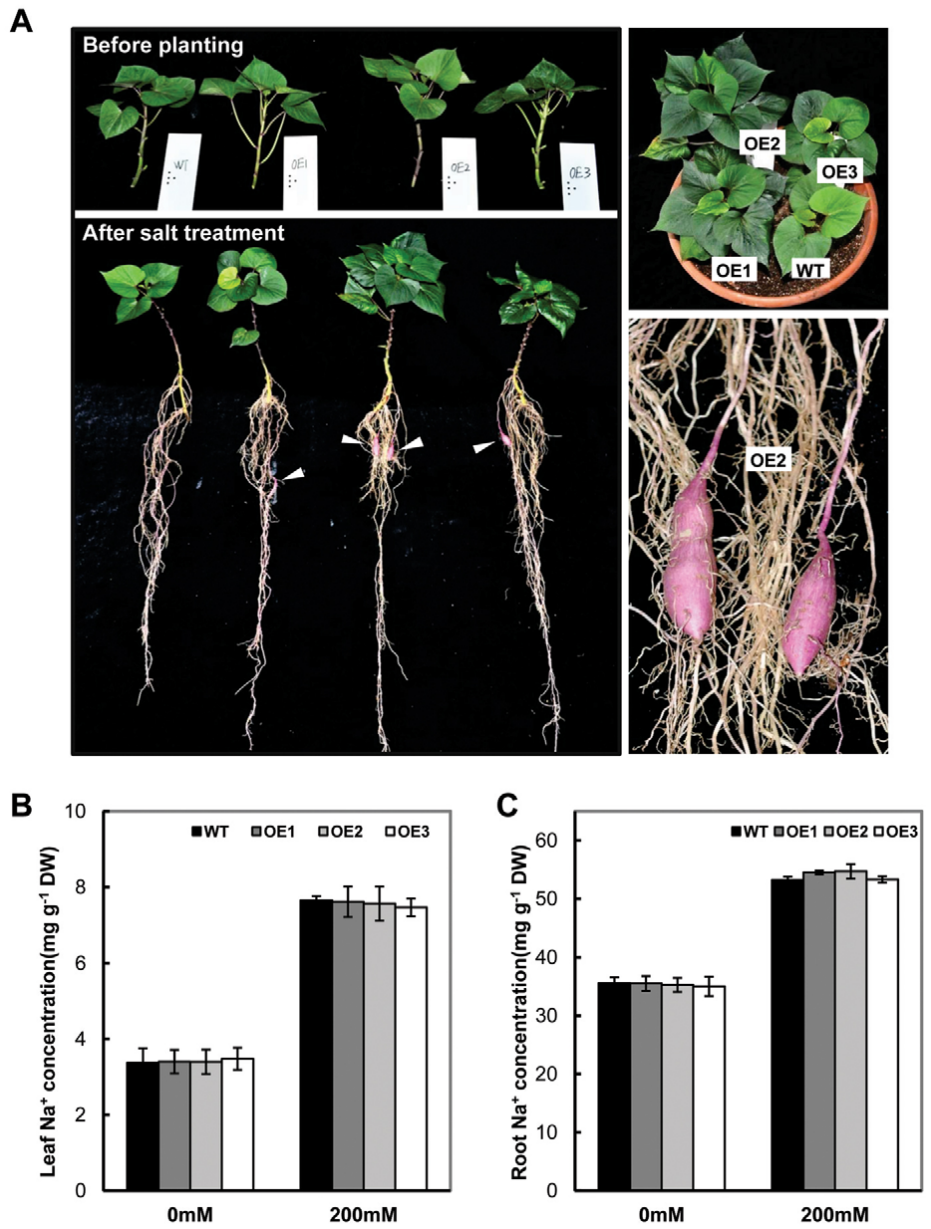
The growth and Na^+^ levels of *SoBADH* transgenic plants under 150 mM salt stress treatment for 6 weeks. A, transgenic OE lines showed distinguishable differences from WT on foliage and root growth after 150 mM salt stress. B–C, Na^+^ level in leaves and roots of WT and transgenic plants. Values represent the mean ± SD (n = 9). Asterisks indicate a significant difference from that of WT at * P<0.05 or ** P<0.01 by *t*-test.

In order to distinguish phenotypic differences between transgenic and non-transgenic plants in the greenhouse, e.g. tuberous root production, the plants were treated with 150 mM NaCl for 6 weeks. The OE plants demonstrated a vigorous growth with more and bigger leaves in comparison with those of WT (Figure 4A, right upper panel). Besides more fibrous roots, the OE plants also developed storage roots. No any tuberous roots were observed in the WT plants (Figure 4A, left lower panel). Among the OE lines, OE2 performed the best with the strongest foliage and biggest storage root development (Figure 3E, right lower panel). After exposure to 150 mM NaCl for 6 weeks, the treatment resulted in a significant accumulation of Na^+^ in the leaves and roots. However, no significant difference was found in the levels of Na^+^ between WT and transgenic plants in both control and treated plants on the basis of leaf and root dry weight (Figure 4B, C).

### Improved tolerance to MV-mediated oxidative stress

**Figure 5:**
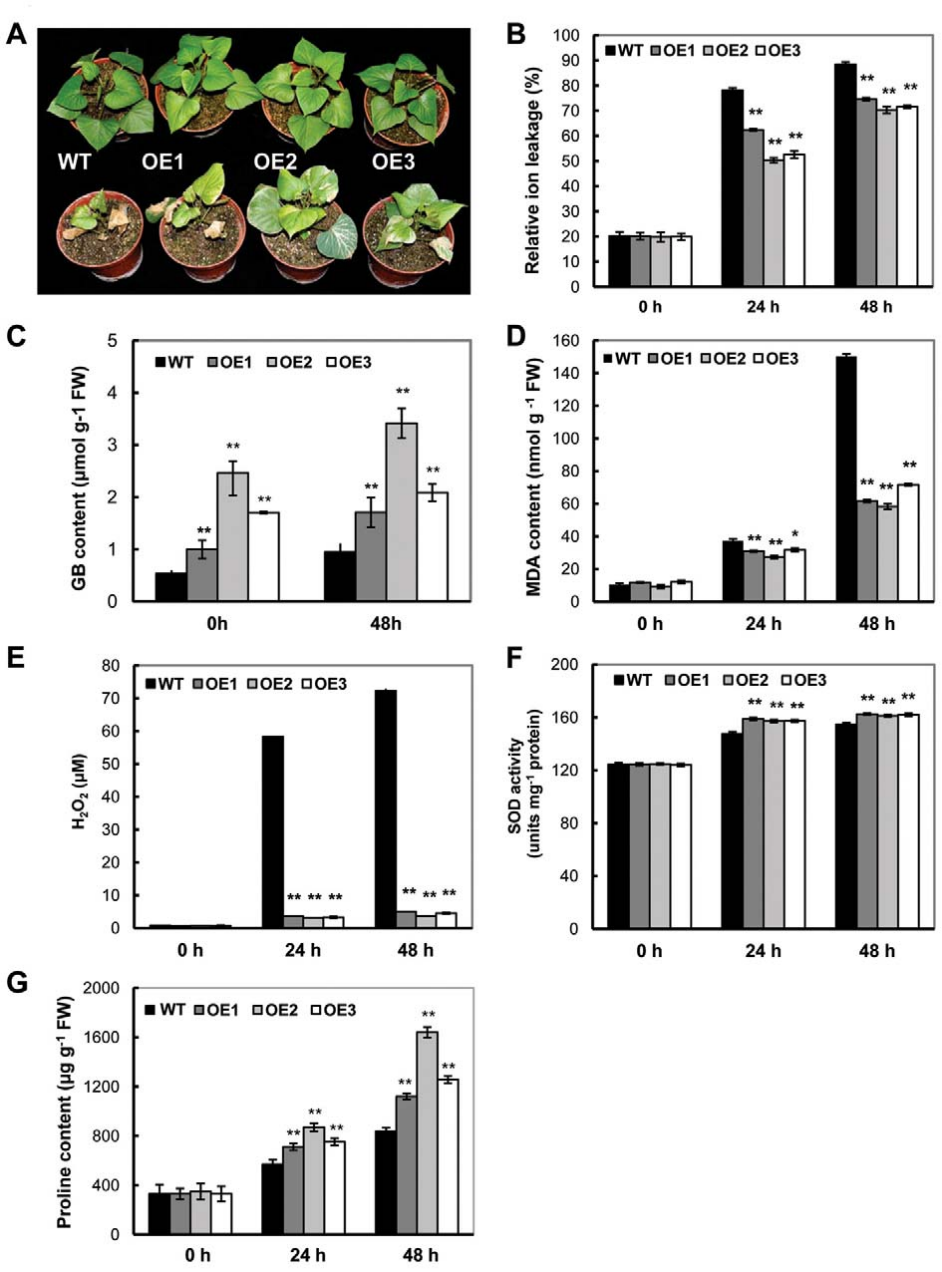
Phenotypic and physiological analyses in the *SoBADH* transgenic plants under MV treatment. A, phenotypic changes before (upper panel) and after (lower panel) MV treatment (photo taken after 5 days); B, decreased ion leaking in the MV-treated leaf discs of the OE plants than that of WT during the time course; C, contents of GB in the leaves of transgenic and WT plants at 48 h after MV treatment; D–E, MDA and H_2_O_2_ contents in the leaves of the transgenic and WT plants at 24 h and 48 h after MV treatment; F, changes in SOD activity in the transgenic and WT plants at 24 h and 48 h after MV treatment. G, proline contents in the leaves of the transgenic and WT plants at 24 h and 48 h after the treatment. Values represent the mean ± SD (n = 9). Asterisks indicate a significant difference from that of WT at * P<0.05 or ** P<0.01 by *t*-test.

Upon treatment with 200 µM MV, oxidative damage was observed in all treated 2-month-old plants. As compared with the WT plants, which showed severe leaf damage, the three OE transgenic plants showed reduced damage (Figure 5A). Consistent with the reduced damage, a significantly reduced ion leakage in the OE transgenic lines was detected (Figure 5B). The GB contents in the three OE plant lines after 48 h of MV treatment were 1.71, 3.4 and 2.1 µmol g^−1^ FW (Figure 5C), respectively, which is significantly higher than that of the treated WT (0.95 µmol g^−1^ FW). During the time course of MV treatment, the proline levels were continuously increased both in the WT and transgenic OE lines. However, the OE lines accumulated a significantly higher amount of proline than the WT. At 48 h, the proline content in the WT reached 837 µg g^−1^ FW, whereas the proline concentration in the OE2 plants was 1640 µg g^−1^ FW, which is a two-fold difference (Figure 5G). Prior to MV treatment, there was no obvious difference of MDA and H_2_O_2_ contents between the transgenic lines and WT (Figure 5D, E). After treatment, the MAD and H_2_O_2_ were decreased in the OE lines as compared with WT. In the time course of MV treatment, the levels of MDA and H_2_O_2_ were elevated in the WT. At 48 h, the MDA and H_2_O_2_ contents in the WT reached 150.3 nmol g^−1^ FW and 72.3 µM, respectively, which is an increase of 14.4 and 87.3 times, respectively. In contrast, the OE lines showed a more reduced accumulation of MDA and H_2_O_2_. For example, the level of MDA and H_2_O_2_ in the OE2 plants was 58.3 nmol g^−1^ FW and 3.7 µM, respectively (Figure 4D, E). The OE2 plants also exhibited a 29.3% increase in SOD activity as compared with WT plants after 48 h of MV treatment (Figure 5F).

### Enhanced tolerance to cold stress

**Figure 6:**
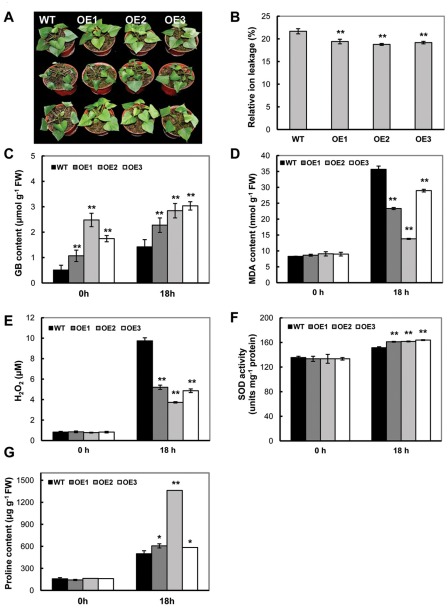
Phenotypic and physiological analyses of the *SoBADH* transgenic plants under cold treatment. A, phenotypic changes before (upper panel) and after (middle panel) cold treatment (4°C for 18 h) and the recovered phenotype (lower panel); B, decreased ion leaking in the cold-treated leaf discs of OE plants than that of WT; C, contents of GB in the leaves of transgenic and WT plants after cold treatment; D–E, MDA and H_2_O_2_ contents in the leaves of the transgenic and WT plants; F, changes of SOD activity in transgenic and WT plants after cold treatment. G, proline contents in the leaves of the transgenic and WT plants after the treatment. Values represent the mean ± SD (n = 9). Asterisks indicate a significant difference from that of WT at * P<0.05 or ** P<0.01 by *t*-test.

To assess the effects of So*BADH* expression on cold stress tolerance in soil-grown plants, 2-month-old plants were incubated for 18 h at 4°C and transferred to 25°C for recovery. When the WT and OE plants were exposed to cold for 18 h, the leaves of the WT plants showed severe wilting and downward curling, whereas those of the OE plants were only slightly affected (Figure 6A). Furthermore, after a 4 h recovery, the OE plants were almost fully recovered to the initial phenotype, whereas the WT plants still exhibited wilting in the leaves (Figure 6A). In addition to phenotypic changes, the levels of electrolyte leakage were also more increased in the WT than in the transgenic lines (Figure 6B). Increased GB expression was detected in the OE lines (Figure 6C), reaching 3.04 µmol g^−1^ FW, which was more than 2-fold greater than that of WT. The low temperature stress also stimulated the production of MDA and H_2_O_2_ in WT leaves (Figure 6F). The levels of MDA in the transgenic lines OE1, OE2 and OE3 only reached to 65.4%, 38.7% and 81.2% of WT, respectively (Figure 6D). The H_2_O_2_ content in these OE plants was accumulated to 53.4%, 38.3%, and 50% of WT, respectively, after 18 h of cold stress (Figure 6E). In response to cold stress, the SOD activity was always higher in the transgenic lines than in WT (Figure 6F). Low temperatures induced more proline accumulation in leaves of the OE lines than those of the WT. After 18 h, the proline content in the leaves of the OE2 plants had increased to 272% (1361 µg g^−1^ FW) of the WT (499 µg g^−1^ FW) (Figure 6G).

### Expression profiling of GB biosynthetic and ROS-scavenging genes

**Figure 7:**
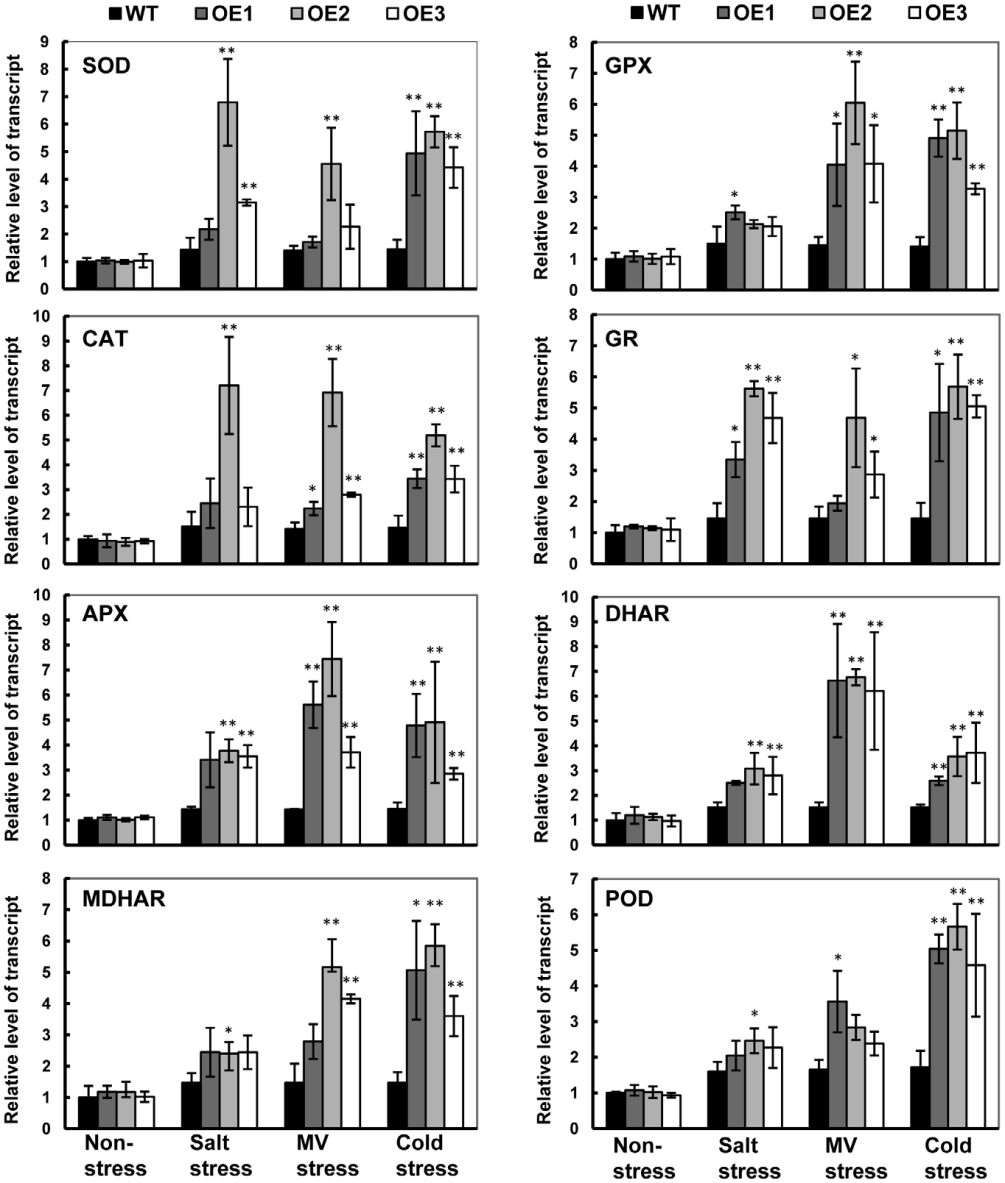
Relative level of transcript of ROS-scavenging genes in wild-type (WT) and *SoBADH* transgenic (OE) plants under stresses. The transcript level of each gene in the leaves under non-stressed condition was used as a reference for comparison. APX, ascorbate peroxidase; BADH, betaine aldehyde dehydrogenase; CAT, catalase; DHAR, dehydroascorbate reductase; GPX, glutathione peroxidase; GR, glutathione reductase; MDHAR, monodehydroascorbate reductase; POD, peroxidase; PSII, photosystem II; ROS, reactive oxygen species; SOD, superoxide dismutase. Values represent the mean ± SD (n = 9). Asterisks indicate a significant difference from that of WT at * P<0.05 or ** P<0.01 by *t*-test.

To investigate the impact of *SoBADH* expression on the gene transcription of native GB biosynthetic genes and stress response pathways, the expression of selected genes (Table S1) was analyzed during the abiotic stress treatments. The most interesting results were obtained from the prevalence of genes encoding the ROS-scavenging enzymes and the genes encoding functions related to the D1 protein and phosphoribulokinase (PRKase). During GB biosynthesis, *SoBADH* expression was significantly increased in the transgenic OE lines in all treatment conditions in contrast with no changes in the level of CMO gene expression (Figure S4). Importantly, a systemic upregulation of the ROS-scavenging enzymes genes, including SOD, catalase (CAT), ascorbate peroxidase (APX), monodehydroascorbate reductase gene (MDHAR), glutathione peroxidase gene (GPX), glutathione reductase gene (GR), dehydroascorbate reductase gene (DHAR) and peroxidase gene (POD), was detected (Figure 7). The transcript levels of the *PRK* and *psbA* genes, which encode phosphoribulokinase (PRKase) and D1 protein, respectively, were not significantly changed in the WT and OE lines under any condition.

## Discussion

This study demonstrates that the expression of the spinach (*Spinacia oleracea* L.) BADH in the sweet potato increases BADH activity and GB biosynthesis in vivo, consequently leading to an enhanced tolerance of the transgenic sweet potato to various abiotic stresses. The increased accumulation of endogenous GB levels also demonstrated an enhanced tolerance, which is consistent with previous studies of the heterologous expression of bacterial choline dehydrogenase (*bet*A), choline oxidase (*cod*A) or the plant *BADH* gene [31], [34], [42], [44], [45]. The elevated GB biosynthesis in sweet potato also improves yield and promote storage root formation. These results indicate that the engineering of enhanced GB biosynthesis in the sweet potato is a feasible approach to improving its tolerance to multiple abiotic stresses.

Many approaches have been used to engineer stress tolerance in plants with limited success [46]. One major problem is due to the undesirable or unfavorable phenotypic changes, such as growth retardation and decrease in yield. So far positive effects of GB accumulation in transgenic plants have been reported, even when grown under non-stress conditions [23], [44]. Even under the stressful conditions, the performance of transgenic plants was better than un-transformed controls, providing great potential for agricultural application [23], [34], [47]. As evidenced in our transgenic OE lines, more than 3-fold increase in the weight of storage roots under normal conditions (Figure 1C) and the tuberous root development under the salt stress (Figure 4A) revealed that the presence of the *BADH* transgene resulted in acceleration of the plant growth, similar to the results reported in Yang *et al.* [48].

**Figure 8:**
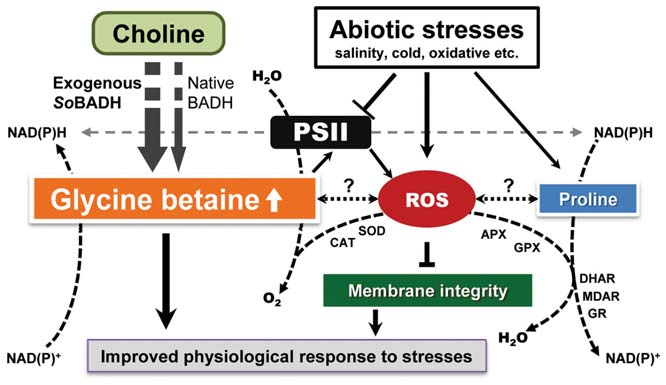
Crosstalks of GB biosynthesis, ROS-scavenging system and proline accumulation under stressful conditions. The overexpression of *SoBADH* directly causes a higher accumulation of GB, in addition to the native GB production, and alters redox homeostasis, leading to more NAD(P)H production, which facilitates proline accumulation and ROS scavenging. GB improves PSII and prohibits ROS generation. Under stress conditions, GB promotes ROS scavenging by the systemic upregulation of ROS-scavenging gene expression, which eventually protects cellular functions and membrane integrity, hence leading to an improved physiological response to the stresses. APX, ascorbate peroxidase; BADH, betaine aldehyde dehydrogenase; CAT, catalase; DHAR, dehydroascorbate reductase; GPX, glutathione peroxidase; GR, glutathione reductase; MDHAR, monodehydroascorbate reductase; POD, peroxidase; PSII, photosystem II; ROS, reactive oxygen species; SOD, superoxide dismutase.

Under stress conditions, sweet potato plants instinctively synthesize and accumulate more GB, as evidenced in our study (a 5-fold increase of GB content in WT under the salt stress, Figure 1B, 2D), indicating that the sweet potato is a GB accumulator. The expression of *CMO* or *BADH* by RT-PCR analysis (Figure S4) also substantiated the hypothesis. A series of physiological studies have proven that GB most likely maintains the osmotic balance between the intracellular and the extracellular environment under salt and drought stress [23], [34], [49]. The levels of GB accumulation in different species are variable, and for certain plants, levels of up to 40 µmol g^−1^ fresh weight of GB have been reported under stress conditions [23], [49]. Under the stress conditions, transgenic OE lines accumulate up to 5.6 µmol g^−1^ FW GB, a minimum of 2 times higher that of WT. In the transgenic sweet potato, such levels of GB could hardly be attributed to the osmotic adjustment to the external environment, especially salinity stress. Nevertheless, a series of studies using transgenic plants have demonstrated that GB, even at low levels, could confer transgenic plants with an increased tolerance to cold, freezing, heat, photo inhibition, drought and salt stress [23], [32], [34]. Mechanistically, GB stabilizes macromolecular activity and membrane integrity [32]. Indeed, a reduced ion leakage and MDA content was detected in the leaf cells of the *SoBADH* transgenic sweet potato (Figures 3D; 5B, D; 6B, D) in our study, suggesting an improved cell membrane homeostasis [31]. Therefore, it can be assumed that GB might protect the cell membrane from stress-induced injuries, likely through alleviating oxidative stress (Figure 8).

Abiotic stresses, such as salinity and low temperature, which are known to disturb redox homeostasis in plant cells, could induce a burst of ROS, resulting in oxidative stress (Figure 8). ROS have been implicated in all types of stresses, and if not scavenged sufficiently, programmed cell death might occur [50], [51]. On the other side, if scavenging too much ROS may negatively affect the signal transduction. Therefore, the balance of ROS generation and scavenging is essential for stress response in plants. Despite GB-mediated enhancement of stress tolerance in many plants, its mechanistic influence on stress signaling and perception remains unclear because of a lack of direct evidence to support GB participation in antioxidative defense systems. Nevertheless, the increased ROS scavenging capacity mediated by GB functions did not negatively affect the signal transduction, as reported by Kathuria H (2009) [52]. They used microarray-based transcriptome to analyses *cod*A transgenic rice and GB accumulation unravelled altered expression of many genes involved in stress responses, signal transduction, gene regulation, hormone signalling and cellular metabolisms. Studies have demonstrated that GB alone does not have antioxidative activity in vitro [53]. Thus, its ROS-scavenging function must be indirect, e.g., via the induction of the synthesis or activation of ROS-defense systems, as illustrated in our proposed model (Figure 8). Such a scenario has been demonstrated both in plants after the exogenous application of GB and in the GB over-produced transgenic plants expressing GB-biosynthetic enzymes [34]. For example, transgenic tomato plants expressing the choline oxidase encoded by the *codA* gene are targeted to chloroplasts, demonstrating an enhanced tolerance against MV-mediated oxidative stress [54]. More profound antioxidant enzyme activity had been evidenced in GB-overexpressing transgenic tomato under stress conditions [55]. Importantly, proline, a typical inert compatible osmolyte that protects subcellular structures and macromolecules under osmotic stress [22], [56], was more accumulated in the OE lines (Figure 3E, 5G, 6G). In GB biosynthesis, the production of NAD(P)H, which is required in proline biosynthesis [56] and ROS scavenging [57], might affect the redox homeostasis and facilitate proline accumulation to activate the ROS-scavenging system (Figure 8).

It is important to maintain a stronger ROS-scavenging ability under stress conditions to alleviate the induced oxidative damage, especially in plant leaves where photosynthesis is dramatically impacted. Plants under abiotic stress have evolved a defense system against oxidative stress by increasing the activity of ROS-scavenging enzymes [58]. Although the oxidative stress was enhanced in the stressed leaves of the sweet potato, as reflected in the increased H_2_O_2_ content, we detected significantly less H_2_O_2_ in the OE transgenic lines as compared with that of the WT (Figure 3B, 5E, 6E). Consistent with this phenomenon, increased SOD expression and activity were detected in the OE lines. In parallel, the expression of other important ROS-scavenging enzymes, including CAT, APX, MDHAR, GPX, GR, DHAR and POD, was also upregulated at the transcriptional level (Figure 7), suggesting that the enhanced tolerance of the transgenic sweet potato plants to abiotic stress is mainly due to improved ROS scavenging (Figure 8). Similar results were also observed in transgenic plants transformed with the *cod*A gene [36]. Thus, in sweet potato plants, it is likely that GB induces an H_2_O_2_-mediated antioxidant system, which includes the enhanced transcriptional expression and activity of the ROS-scavenging enzymes.

It has been reported that GB protects the cells from stresses by stabilizing the quaternary structures of complex proteins like antioxidant enzymes and biomembranes of other functional units, such as the oxygen-evolving PSII complex [32], [59]. Previous studies suggest that the main effect of ROS is the inhibition of the repair of photodamaged PSII by the suppression of de novo protein synthesis; the primary sites of photodamage are the oxygen-evolving complex and the D1 proteins [60]. The rate of repair of photodamaged PSII is severely depressed by various types of stress, such as oxidative stress [43], [61], [62], salt [63], [64] and low temperature [62], with subsequent increases in the extent of photoinhibition. The effect conferred by GB has been shown to protect the machinery required for the degradation and synthesis of the D1 protein under stress exerted by high-salt conditions [65], [66]. In addition, the chloroplast-targeted production of GB in transgenic tomato plants protects photosynthetic machinery more efficiently than the cytosol-targeted production, showing the accumulation of GB in chloroplasts is more effective than that in the cytosol for the protection of plants against abiotic stress [55]. In our study, the OE plants maintained higher Fv/Fm values over the entire period of salt stress (Figure 2E). However, the results from the analysis by real-time PCR revealed that the transcriptional levels of the *psbA* and *PRK* genes, which encode the D1 protein and PRKase, respectively, were not significantly affected by the stresses or overexpression of GB in the plant cells. These results suggest that, under stressful conditions, the functions of these genes might work or regulate at the protein level. We suppose that the accumulation of GB in the transgenic sweet potato might stabilize the conformation of PRKase and maintain the enzyme in a functionally active state under salt stress, acting as a molecular chaperone.

A few reports have proposed the GB stabilizes the highly ordered structures of certain complex proteins to prevent denaturation when plants or plant cells are exposed to stress conditions [59], [67]. Based on our study, we propose that elevated GB biosynthesis could induce or promote the expression of certain stress-responsive genes, e.g., enzymes that scavenge ROS and proline biosynthesis, and subsequently reduce ROS accumulation in plant cells, resulting in the protection of the photosynthetic machinery from the damage caused by abiotic stresses (Figure 8).

In conclusion, GB accumulation in the transgenic sweet potato by the heterologous overexpression of the *SoBADH* gene dramatically improved the tolerance to salt, oxidative and cold stresses mediated by induction or activation ROS scavenging, increased proline accumulation and improved protection of membrane integrity. Thus, this study has increased our understanding of the molecular mechanisms of GB function in plants, although some aspects are still unclear (Figure 8), such as how ROS affects the biosynthesis of GB and proline at the molecular level. Nevertheless, the enhanced performance of these transgenic sweet potatoes without phenotypic defects proves that engineering the GB biosynthetic pathway is not only a feasible approach to improving the economically important crop but also demonstrates great promise for the breeding programs of other crops.

## Materials and Methods

### Plasmid construction and production of transgenic sweet potato

The expression cassette containing the CaMV35S promoter-driven *BADH* gene from *Spinacia oleracea* targeted to chloroplasts [40], [48], [54] was cloned into the pCAMBIA1301-based plant expression vector to generate the binary vector pCSoBADH (Figure S1). The pCSoBADH was introduced to *Agrobacterium tumefaciens* strain LBA4404 for the sweet potato transformation. Embryogenic calli of sweet potato cultivar Sushu-2 were used as explants for transformation. The induction, propagation and plant regeneration of embryonic callus were performed as described by Yang et al. [13]. Four-week-old embryogenic suspension cultures were used to inoculate LBA4404 harboring the pCSoBADH for the production of transgenic plants in accordance with the protocol described by Yang et al. [13]. To eliminate escapes, putative plants were screened for rooting under the selection with 10 mg l^−1^ hygromycin in a basic sweet potato shoot-culturing medium.

### Molecular characterization of transgenic sweet potato

Genomic DNA was isolated from in vitro-cultured plants using the method described by Kim and Hamada [68]. For the polymerase chain reaction (PCR) detection, 300 ng of the genomic DNA was used as the template. The specific primers 5′-TTCTACACAGCCATCGGTCC -3′ and 5′- AGGCTCTTCGAGCTGCCACCAC -3′ were used to amplify a 500 bp fragment of the hygromycin phosphotransferase (HPT) gene. PCR was performed with an initial denaturation step at 95°C for 4 min; followed by 30 cycles at 95°C for 30 s, 58°C for 30 s, and 72°C for 30 s; and a final extension at 72°C for 10 min. The PCR products were separated by electrophoresis on a 1% (w/v) agarose gel.

For Southern blotting, 20 µg of genomic DNA was digested with *Eco*RI, which is a unique restriction enzyme site within the full-length T-DNA region. The digested products were purified and fractionated by 0.8% agarose gel, subsequently transferred to an Amersham Hybond N^+^ nylon membrane (GE Healthcare Life Sciences, Indianapolis, IN) by capillary transfer. A 1 kb DIG-dUTP labeled fragment of So*BADH* gene was amplified using the primers 5′-GCCACATACTTGCGTGCTATTGCTGC-3′ and 5′-CAATGCAATGGCTTCATCTTCGGAAC-3′ and a PCR DIG Probe Synthesis Kit (Roche Applied Science, Germany). The hybridization and detection were performed according to the manufacturer's instructions, using the DIG-High Prime DNA Labeling and Detection Starter Kit II (Roche Applied Science, Germany).

The expression of So*BADH* mRNA was analyzed by reverse transcriptase PCR (RT-PCR). Total RNA was extracted from leaf tissue using RNAprep Pure Plant kit (Tiangen, Beijing, China) following the manufacturer's instructions, treated with DNase and reverse transcribed using M-MLV Reverse Transcriptase RNaseH (Toyobo, Japan). The following primers were used for the RT-PCR analysis: So*BADH*-specific primers (5′- ATGGCGTTCCCAATTCCTGCTC-3′, 5′- AGGCTCTTCGAGCTGCCACCAC-3′) were used to amplify a 172 bp product, and actin gene-specific primers (5′- GCCATTCAGGCTGTTCTCTC-3′, 5′- GGCTGTGGTGGTGAAAGAGT-3′) were used as an internal control. The leaves of putative transgenic plants were tested for β-glucuronidase expression using the histochemical GUS assay according to Jefferson et al [69].

### Phenotypic analysis of transgenic sweet potato

The 2-month-old shoots of non-transformed control (wild-type) and *SoBADH* over-expressing (OE) plant lines were transplanted to plastic pots (diameter, 15 cm) containing a mixture of vermiculite, turf and humus (1∶1∶1, v/v/v) in the greenhouse, with one plant per pot. Each line was repeated a minimum of three plants. All pots were watered sufficiently with half-Hoagland solution every 5 days. After 5 months, the plant status and the biomass of the storage roots were recorded. The experiment was repeated three times.

### NaCl, MV and cold treatments

For the salt treatment, 2-month-old wild-type (WT) and transgenic plants in 15-cm diameter pots were irrigated with a 200 ml of 200 mM NaCl solution once every 2 days for 16 days and then with tap water for 4 days in a plant growth chamber at 25°C. The fresh weight of whole plants was measured immediately after the treatment in comparison with plants grown under normal conditions. For the MV treatment, 2-month-old plants were sprayed with 200 µM of MV solution once every 2 days. The MV was dissolved in a 0.1% Tween-20 solution and sprayed on the tops of the leaves of the tested plants. Leaf damage caused by MV was recorded 5 days after treatment. For the cold treatment, the sweet potato plants were transferred to a refrigerated growth incubator (Friocell404, MMM Medcenter Einrichtungen GmbH, Germany) and maintained at 4°C for 18 h. Following the stress treatment, the plants were transferred back to normal culture conditions (25°C) for recovery. All treatments were performed in triplicate.

### Growth of sweet potato plants in greenhouse under salt stress and determination of total Na^+^ contents

The 2-month-old shoots at the same stage were transplanted in plastic pots (diameter, 35 cm; height, 25 cm) containing a mixture of vermiculite, turf and humus (1∶1∶1; v/v/v) in the greenhouse. Each pot contained one WT plant and the three different lines (OE1, OE2 and OE3). After irrigation with half-Hoagland solution every 5 days for 4 weeks, the plants were watered with half-Hoagland solution containing 150 mM NaCl. The plants were irrigated at 5 day intervals up to 6 weeks. Then, the plants were sampled by careful washing off of the soil with water. The roots and shoots of the plants were harvested at the end of the salt treatment. Dry weight was measured after 48 h incubation at 80°C. Plant samples were digested with HNO_3_, and Na^+^ contents were determined by ion selective electrode method using an ion analyzer (PXSJ-216, Leici, Shanghai, China). The treatment was performed in triplicate.

### Ion leakage analysis of leaf discs

For analyzing the MV damage on plant leaves, thirty leaf discs (6 mm diameter) from the third leaves of 2-month-old plants grown in soil were floated on a solution containing 0.4% (w/v) sorbitol and 5 µM MV. The samples were incubated in darkness for 12 h to allow for MV diffusion and then subjected to continuous light (150 µmol m^−2^ s^−1^) at 25°C. The ion leakage was assessed using an ion conductivity meter (FE30, Mettler Toledo, Switzerland) over a period of 0 to 48 h. At the end of the specified time period, the samples were boiled for 10 min to release the solutes. The conductivity of the solution was measured again, and this value was considered 100% ion leakage in calculations of the relative ion leakage at different time points. The treatment was performed in triplicate.

For evaluating cellular damage under freezing stress, twenty leaf discs (6 mm diameter) collected from the third leaves of two-month-old plants were assayed by measuring leakage of electrolytes through membranes as described by Huang et al. [70]. The leaf discs were placed in individual glass tubes and incubated in a refrigerated circulator at −1°C for 1 h, and the temperature was then decreased to −5°C for 2 h and thawed overnight at 4°C. Deionized water (10 ml) was added, and the tubes were incubated at 25°C for 6 h before measuring the electrical conductivity. Another conductivity measurement was taken after freezing the samples at −70°C and then thawing; this measurement represents the total membrane leakage. The ratio of the two measurements represents the relative damage. The extent of cellular damage was quantified by the level of ion leakage, a typical indicator of membrane disruption. The treatment was performed in triplicate.

### Measurement of GB content by ^1^H NMR

The sweet potato leaves (300 mg) were frozen in liquid nitrogen, ground into a powder and thawed to extract the sap. The extracted leaf sap was centrifuged at 12,000 *g* at 4°C for 10 min, and the supernatant was dried under a stream of nitrogen gas. The dried fractions were dissolved with 500 µl D_2_O, and 50 µl 10 mmol l^−1^ 3-(trimethylsilyl)-propane-sulfonic acid sodium salt (DSS) was added as internal reference for quantification. The GB content in the leaves of WT and transgenic plants was determined using ^1^H NMR spectroscopy as described by Quan et al. [37].

### Measurement of chlorophyll and proline content

The sweet potato leaf samples (200 mg) were homogenized with 95% ethanol (v/v), and incubated under dark conditions for 2 days. The homogenate was filtered through a filter paper. The total chlorophyll content was determined spectrophotometrically according to methods described by Arnon [71]. Proline was extracted from 0.1 g freeze-dried leaf samples and spectrophotometrically determined using the acid ninhydrin method published by Hanson et al. [72].

### Determination of lipid peroxidation

The lipid peroxidation in the leaf tissues was assayed in terms of malondialdehyde (MDA) in the samples according to the published methods of Dhindsa and Matowe [73]. The leaves (200 mg) from the sweet potato plants were homogenized in 5 ml of 10% trichloroacetic acid (TCA) and centrifuged at 12,000 *g* for 10 min. Two milliliters of clear supernatant were added to 2 ml of 0.6% thiobarbituric acid (in 10% TCA), and the reaction mixture was incubated at 100°C in a water bath for 15 min. The reaction was terminated at room temperature, and the reaction mixture was centrifuged at 12,000 *g* for 10 min. The absorbance of the supernatant at 450, 532, and 600 nm was determined with a spectrometer, respectively. The concentration of MDA was calculated by the following formula: C (µmol l^−1^) = 6.45×(OD_532_–OD_600_)-0.56×OD_450_

### Qualitative and quantitative analysis of H_2_O_2_

To visualize H_2_O_2_ production, the sweet potato leaves were placed in 1 mg ml^−1^ of 3, 3-diaminobenzidine (DAB) solution, pH 3.8. The samples were subsequently incubated for 6 h in a growth chamber, and the Chl was removed at 80°C for 2 h in 80% ethanol. The H_2_O_2_ content was assessed according to Sairam and Srivastava [74]. The concentration of H_2_O_2_ was estimated by measuring the spectrum absorbance of the titanium-hydroperoxide complex and using a standard curve plotted with known concentrations of H_2_O_2_.

### Assay of SOD and PSII activity

To assess SOD activity, a fine powder of sweet potato leaves (about 200 mg) was resuspended in 50 mM potassium phosphate (pH 7.8) containing 0.1 mM EDTA and 1% PVP and then centrifuged at 12,000 *g* for 15 min at 4°C. For the assay, 3 ml of reaction mixture containing 50 mM potassium phosphate (pH 7.8), 0.1 mM EDTA, 1% PVP, 13 mM methionine, 75 µM nitro blue tetrazolium (NBT), and 30 µl of enzyme extract was added to 2 µM of riboflavin. One unit of SOD activity was defined as the amount of enzyme required to inhibit 50% NBT photoreduction. The mixtures were illuminated in glass test tubes for 10 min, and the absorbance of the mixtures at 560 nm was quickly determined with a spectrometer. The SOD activity was expressed as unit mg^−1^ of protein.

The photosynthetic activity was recorded via chlorophyll fluorescence determinations of the photochemical yield (Fv/Fm), which represents the maximum quantum yield of PSII, using an imaging chlorophyll fluorometer (Walz Imaging PAM, Walz GmbH, Effeltrich, Germany) after a 30 min dark adaptation. The measurements were conducted at room temperature (25°C) using saturated light flashes on the fifth leaves of the plants.

### Analysis of BADH activity

The fresh sweet potato leaf samples (500 mg) from the transgenic and control plants were ground to a powder in liquid nitrogen and homogenized in 1 ml of extraction buffer containing 50 mM 4-(2-hydroxyethyl)- 1-piperazineethanesulfonic acid (HEPES)-KOH (pH 8.0), 1 mM ethylene diamine tetraacetic acid (EDTA), and 5 mM dithiothreitol (DTT). The plant debris was removed by centrifugation at 10,000 *g* for 10 min at 4°C. The protein concentration of the supernatant was determined using the Bradford method [75]. BADH activity was assayed independently by the betaine aldehyde-specific reduction of NAD^+^ at 22°C [76]. The reactions were carried out in a final volume of 1 mL containing 50 mM HEPES-KOH (pH 8.0), 10 mM EDTA, 1 mM NAD^+^, 1 mM betaine aldehyde and 1 mg protein extract. One unit of BADH equals 1 nmol NAD^+^ reduced min^−1^ mg^−1^protein.

### Transcriptional expression of stress relate genes

For the quantitative real-time reverse transcription polymerase chain reaction (qRT-PCR), total RNA extracted from the sweet potato leaves after stress treatment using the RNAprep Pure Plant kit (Tiangen, Beijing, China) was treated with DNase and reverse transcribed with M-MLV Reverse Transcriptase RNaseH (Toyobo, Japan). Gene-specific primers of sweet potato (Table S1) were used for determining the expression levels of genes using the SYBR Green PCR master mix (Toyobo) in a Bio-Rad CFX96 thermocycler. The amplification conditions were 95°C for 1 min, followed by 40 cycles of 95°C for 15 s and 60°C for 30 s. The actin gene was used as the internal control.

### Statistical analysis

All data were represented as mean ± SD from at least three independent experiments with three replicates each. Statistical significances of the differences were determined by Student's *t*-test. The difference between treatments was considered as significant when *P*<0.05 or 0.01 in a two-tailed analysis.

## Supporting Information

Figure S1 **Schematic representation of the T-DNA region of pCSoBADH harboring the** ***SoBADH-*** **,** ***HPT-*** **and** ***uidA*** **-expressing cassettes.**
(TIF)

Figure S2 **Analysis of GUS activity in the leaves of transgenic sweet potato plants (OE1–OE9) showing the transgenic nature by dark-blue and non-stained wild-type plants (WT).**
(TIF)

Figure S3 **Molecular characterization of the** ***SoBADH*** **transgenic sweet potato plants.**
(TIF)

Figure S4 **Relative expression of the** ***BADH*** **,** ***CMO*** **,** ***PRK*** **and** ***psbA*** **genes in the leaves of wild-type (WT) and** ***SoBADH*** **transgenic (OE) plants under the NaCl, MV and cold treatments.**
(TIF)

Table S1 **Primers used for gene amplification by real time RT-PCR.**
(DOCX)
