# Supplementary figures and images for: Improved Tolerance to Various Abiotic Stresses in Transgenic Sweet Potato (Ipomoea batatas) Expressing Spinach Betaine Aldehyde Dehydrogenase
Source: PLoS One. 2012 May 16;7(5):e37344. doi: 10.1371/journal.pone.0037344 (PMC3353933; doi:10.1371/journal.pone.0037344)

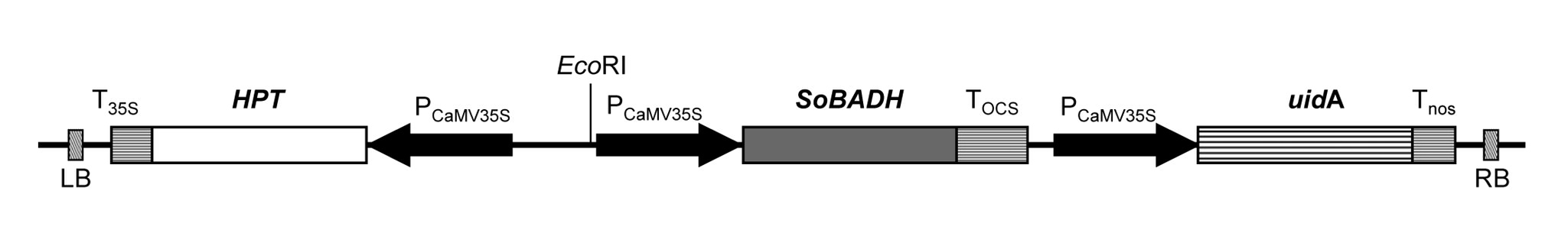

Supplement: Figure S1 — Schematic representation of the T-DNA region of pCSoBADH harboring the SoBADH- , HPT- and uidA -expressing cassettes. (TIF) [file pone.0037344.s001.tif]

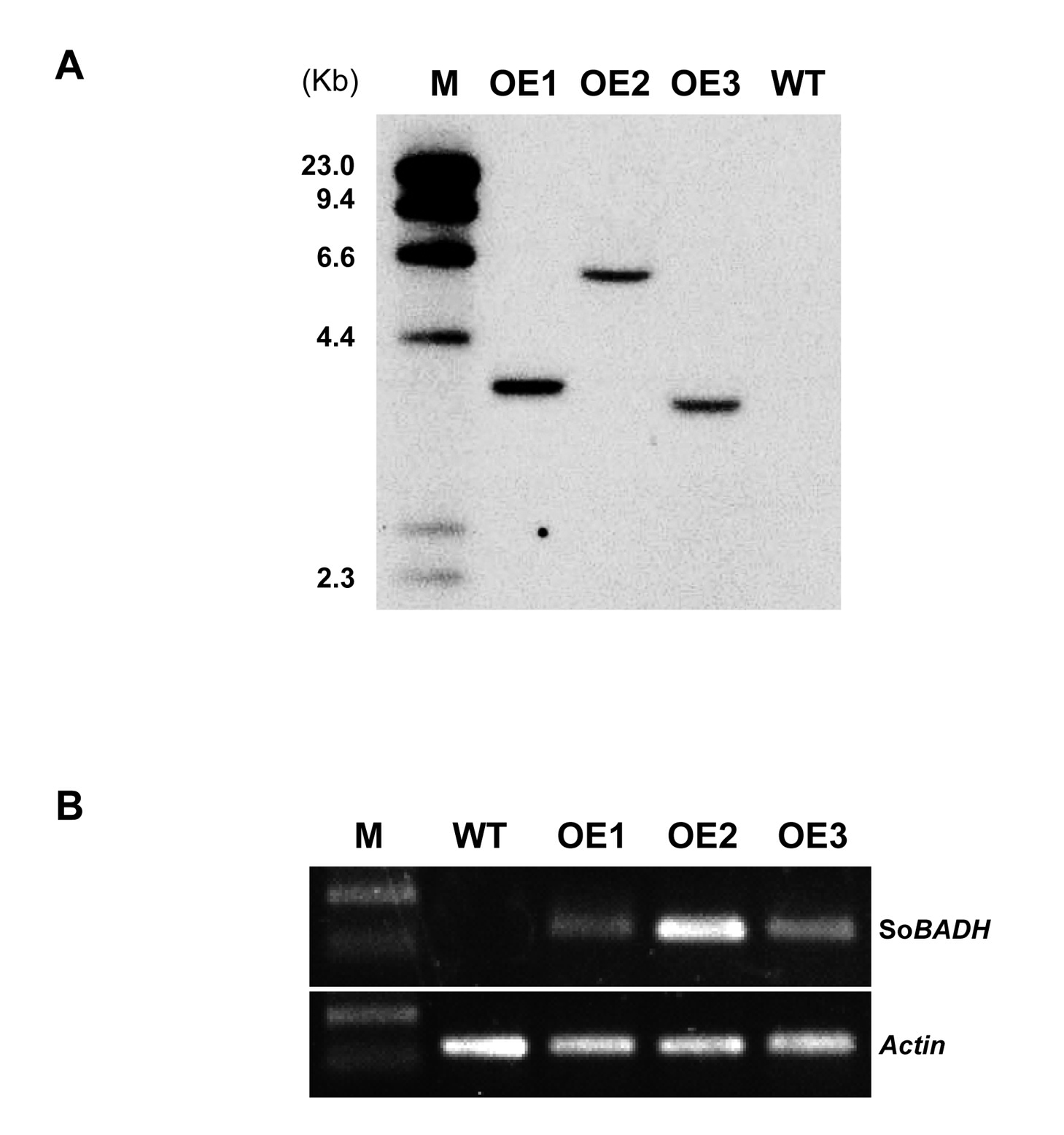

Supplement: Figure S3 — Molecular characterization of the SoBADH transgenic sweet potato plants. (TIF) [file pone.0037344.s003.tif]

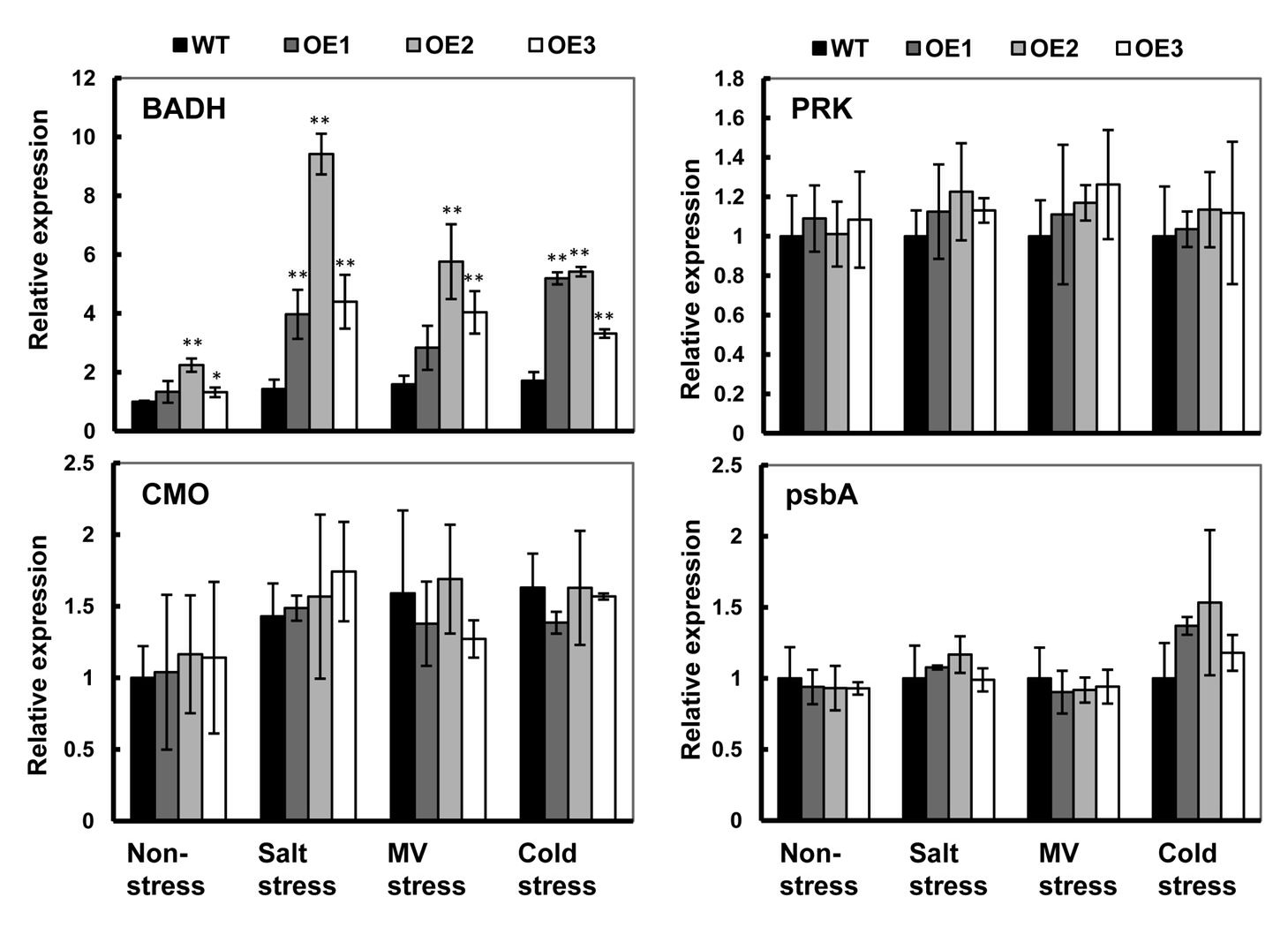

Supplement: Figure S4 — Relative expression of the BADH , CMO , PRK and psbA genes in the leaves of wild-type (WT) and SoBADH transgenic (OE) plants under the NaCl, MV and cold treatments. (TIF) [file pone.0037344.s004.tif]
