# Supplementary material for: Improved Tolerance to Various Abiotic Stresses in Transgenic Sweet Potato (Ipomoea batatas) Expressing Spinach Betaine Aldehyde Dehydrogenase
Source: PLoS One. 2012 May 16;7(5):e37344. doi: 10.1371/journal.pone.0037344 (PMC3353933; doi:10.1371/journal.pone.0037344)
Supplement: Table S1 — Primers used for gene amplification by real time RT-PCR. (DOCX) [file pone.0037344.s005.docx]

| **Gene** | **Primer** | **Sequence (5'- 3')** | **Product size (bp)** |
| --- | --- | --- | --- |
| **Actin** | Actin-F | TTCCGATCTCTCTCGCACTC | 210 |
|  | Actin-R | CCTCTTCTGCCATCTTCTGC |  |
| **APX** | APX-F | CCTGCTGGTCATTTACGTGA | 150 |
|  | APX-R | CTGGCCCATCTTTGGTGTAT |  |
| **BADH** | BADH-F | CCGCTACTGAACAAGTCATT | 150 |
|  | BADH-R | TGCAGCAATAGAACGCAAAT |  |
| **CAT** | CAT-F | ACGCAATTCCCGGACGTGAT | 170 |
|  | CAT-R | AAGCCTTCCATGTGGCGGTA |  |
| **CMO** | CMO-F | TGCATCATTTCCACTGCCTA | 149 |
|  | CMO-R | CTTCACCCATCAAGAAAGAG |  |
| **DHAR** | DHAR-F | TGTGTCAAGGCTGCTACTGG | 148 |
|  | DHAR-R | TTGCCTTCAGGAACCATTCA |  |
| **GPX** | GPX-F | GAACAGGGAAGGAAAGGTTG | 150 |
|  | GPX-R | TCTGAAACTTGGTGCTTCCA |  |
| **GR** | GR-F | TTGCAGTTGCTTTGAGAGCT | 148 |
|  | GR-R | TCAGACTCCGCTGTTCCCTC |  |
| **MDHAR** | MDHAR-F | CTACTCCCGTGCCTTTGATT | 151 |
|  | MDHAR-R | CTCCAAGAATGCACCAACAA |  |
| **POD** | POD-F | TTCACGACTGCTTCGTTGA | 149 |
|  | POD-R | TTCTCAACCGCGGTCTTAA |  |
| **PRK** | PRK-F | GCTCTCAACATAGATCAGCT | 167 |
|  | PRK-R | TGAAGGCTCTACTATCTCAT |  |
| **psbA** | psbA-F | CATCCGTTGATGAATGGTTA | 157 |
|  | psbA-R | GCAACAGGAGCTGAGTATGC |  |
| **SOD** | SOD-F | TCCTGGACCTCATGGATTTC | 150 |
|  | SOD-R | GCCACTATGTTTCCCAGGTC |  |

**Notes:** APX, ascorbate peroxidase; BADH, betaine aldehyde dehydrogenase; CAT, catalase; CMO, choline monooxygenase; DHAR, dehydroascorbate reductase; GPX, glutathione peroxidase; GR, glutathione reductase; MDHAR, monodehydroascorbate reductase; POD, peroxidase; PRK, phosphoribulokinase; psbA, photosystem II protein D1; SOD, superoxide dismutase.
